# Female Sexual Function Index Outcome After Posterior Vaginal Tightening Approach and Anterior Cervical Ring Repair when Indicated

**DOI:** 10.1007/s00266-025-04899-5

**Published:** 2025-05-16

**Authors:** Emre Köle, Bertan Akar, Alparslan Deniz, Merve Çakır Köle, Erdoğan Aslan, Eray Çalışkan

**Affiliations:** 1 Department of Obstetrics and Gynecology, Alanya Alaaddin Keykubat University School of Medicine, Oba Mahallesi Fidanlık Cd. 07400, Alanya, Antalya Turkey; 2 Department of Obstetrics and Gynecology, Private Medar Hospital, Yeniköy Merkez, Mine Çiçeği Sokak No:6, 41275 Başiskele, Kocaeli Turkey; 3 Department of Obstetrics and Gynecology, Private Practice, Oba Mahallesi Fidanlık Cd. 07400, Alanya, Antalya Turkey; 4 Department of Obstetrics and Gynecology, Private Practice, İzmit, Kocaeli Turkey

**Keywords:** Female sexual function index, Vaginal laxity, Cervical ring repair, Posterior vaginal tightening

## Abstract

**Background:**

Female sexual dysfunction is believed to be associated with pelvic floor dysfunction in most cases. However, correcting prolapse does not always necessarily correct sexual function. The reason for this might be secondary to disregarding anatomically relevant structures during surgical interventions. We aimed to demonstrate that posterior vaginal tightening approach avoiding anteriorly located structures, such as clitoral complex, would yield better results in terms of sexual function.

**Methods:**

Fifty-seven postmenopausal women with primary complaints of vaginal laxity and Grade I and II prolapse were operated. All patients received posterior vaginal tightening operation, and a cervical ring repair was utilized when indicated (n:25). Perineal repair was done if there was any defect (n:13). Levator plication is not done in any patients. FSFI (Turkish Version) was applied to each patient prior to surgery and at 6th month postoperatively. A Likert-type scale is also utilized to assess the patient satisfaction from the procedures.

**Results:**

All the domains and the total score of FSFI were observed to be improved. Only the improvement in the pain domain scores was not statistically significant. Satisfaction of the patients from the surgery on a Likert scale was so as to: very satisfied 27 (47.4%), satisfied 12 (21.1%), neither satisfied nor dissatisfied 8 (14%), dissatisfied 5 (8.8%), very dissatisfied 1(1.7%).

**Conclusion:**

Sexual function of women with vaginal laxity can be improved when vulvovaginal erotogenic complex is not disrupted. This can be achieved via a posterior approach while maintaining successful anatomic correction of both posterior and anterior compartments.

**Level of Evidence III:**

This journal requires that authors assign a level of evidence to each article. For a full description of these Evidence-Based Medicine ratings, please refer to the Table of Contents or the online Instructions to Authors www.springer.com/00266.

## Introduction

Female sexual dysfunction (FSD) is believed to be effecting approximately 1/3 to ½ of women of all ages [1–3]. In a study done by Dietz et al., it was stated that almost 24% percent of all women who were referred to urogynecology clinics had the symptoms or signs of vaginal laxity [4]. Given the magnitude of the problem, the matter is further complicated by its multidimensional negative effects on the quality of life of women and unequivocal scientific data regarding the nature and treatment of this prevalent problem. FSD is defined as disorders of desire, arousal, orgasm, and pain that lead to marked distress in the emotional well-being of the woman. The causes of FSD may involve social, psychological, or physical problems. Nevertheless, pelvic floor disorders such as pelvic organ prolapse (POP) or urinary incontinence (UI) are reported to be the major causative factor [5–7]. Once the POP or UI is addressed, one can assume that the symptoms associated with these problems would also be alleviated accordingly. However, there are controversial reports in the literature. While some authors claim that repairing pelvic organ prolapse makes no improvement in some aspects of female sexual function [8], others report significant improvements with surgical interventions [9]. There are also some reports in which even the overall sexual function is believed to be improved, and there are detrimental effects on orgasm and orgasm intensity and an increase in pain domains [10, 11]. The authors of this study believe that the reason for these discrepant reports in the literature originates from the fact that women’s sexuality is still poorly understood and surgeries are done without paying special attention to the physiological, anatomical, and biochemical machinery of female sexuality.

The initial reports of Kinsey et al. and Masters and Johnson at the beginning of the second half of the twentieth century on female sexual function were based on observations of male sexual response [12]. Today there are still controversies about female orgasm. Janini et al. support the notion of two distinct types of orgasms. The first type of female orgasm was obtained through the direct stimulation of the external clitoris, therein referred as clitorally activated orgasm (CAO). The second type was described as that obtained during stimulation of the vagina with penile thrust. This type of orgasm was proposed to be named as vaginally activated orgasm (VAO). The anatomical structures that might provoke VAO or CAOs have not been completely described and probably represent a unique case of major uncertainty regarding human gross anatomy [12]. After the publication of the book “The G-spot and other discoveries about human sexuality” [13], the focus of both scientific societies and media began to be intensified on the anterior vaginal wall. On the other hand, Puppo in his excellent review clearly stated that there is no vaginal orgasm or G-spot [14]. In fact, whether their nomenclature or origin of orgasm is different from each other or not, both ecoles highlight the presence of anatomically interrelated and functionally efficient cooperation of various female genital organ parts. While Jannini et al. advocate the role of a clitero-uretro-vaginal complex, Puppo proposed to name the female erectile tissues as female penis. Anatomic studies supported that biopsies taken from the anterior vaginal wall were more densely innervated than samples from the posterior wall [15]. Furthermore, it was also reported that distal parts of the vaginal wall were thicker and had a greater number of nerve fibers than proximal regions [15, 16]. Hence, O’Connel et al. noted that “the distal vagina is a structure that is so interrelated with the clitoris that it is a matter of some debate whether the two are truly separate structures” and coined the term “clitoral complex” to reflect this concept. A step forward, they proposed that although the distal vagina and the urethra are not erectile tissues, these structures are intimately related to the bulbs and cavernous bodies of the clitoris. The three structures share the same vasculature and nerve supply and respond as a unit during sexual stimulation [17]. There are also some studies demonstrating that biochemical machinery mediating peripheral excitatory signaling was expressed in tissues surrounding the distal regions of both the vagina and urethra. Demonstration of cells in the lining of blood vessels rich in phosphodiesterase type 5 [18], the presence of nitric oxide synthase expression in nerve fibers [19], and the presence of exocrine glands around the urethra, capable of producing prostate-specific antigen [20–22], can all be considered as further evidence. These studies further support the pivotal role of the vagina in that CUV complex plays a key role in female sexual response.

Unfortunately, surgical approaches that correct pelvic floor disorders largely ignore the uprising concepts that shed light on female sexuality. Hence, it is of no surprise that the reports concerning the sexual outcomes produce conflicting results. In our study, we tried to preserve the so-called CUV complex and used a posterior approach to correct vaginal laxity and presented our results here.

## Materials and Methods

During the study period, 248 cases were operated for stage 1 to 4 pelvic organ prolapsed [23], and 71 cases were operated for stress urinary incontinence using retropubic tension-free vaginal tape procedure. After excluding stage 3–4 POP-Q cases and pure stress urinary incontinence cases, 66 cases with primary complaints of vaginal laxity were evaluated. After discussing alternative therapies, nine postmenopausal cases received vaginal laser therapy and the remaining 57 premenopausal sexually active cases decided to undergo vaginal tightening operation. Each patient was individually assessed.

### Surgical Technique

**Fig. 1:**
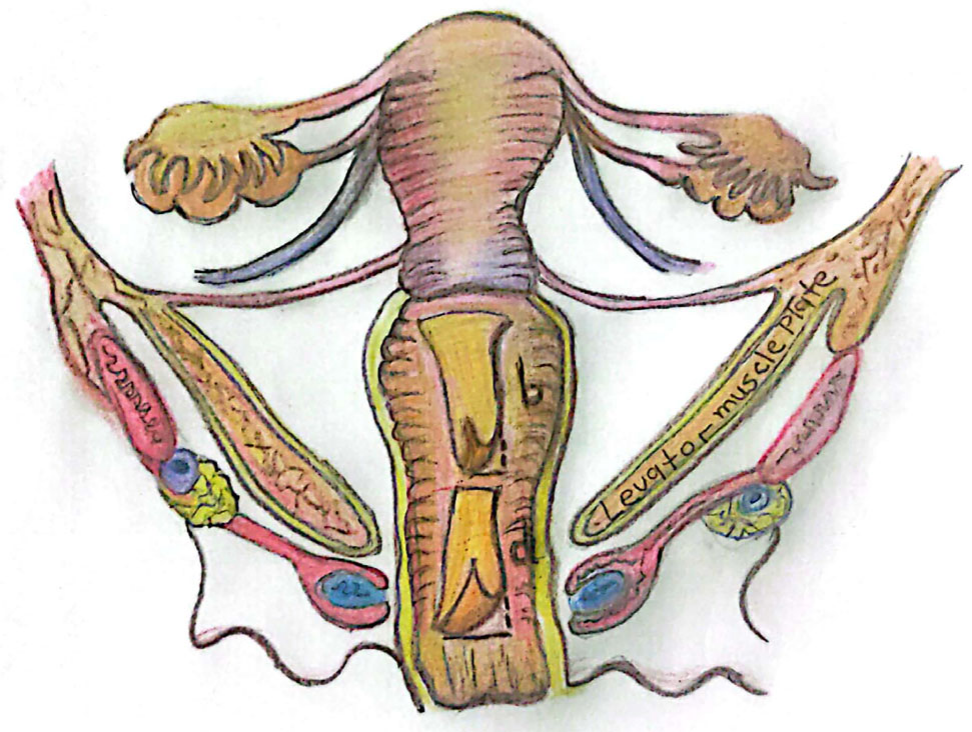
**a** Proximal vaginal excision **b** Distal vaginal excision

All of the operations were done by the same surgical team lead by same operator (EC). The patients were assessed preoperatively for signs and symptoms of pelvic floor dysfunction, and vaginal mapping was done to identify the areas of laxity. The vaginal laxity was grouped as anterior wall laxity, posterior wall laxity, and uterine prolapse with sacrouterine ligament laxity. Posterior vaginal wall is divided into two areas with reference to the levator muscle plate (Fig. 1). Distal vagina is defined as the area below the levator muscle plate down to the vaginal introitus. Likewise, the proximal vagina is also defined as the area above the levator muscle plate up to the posterior fornix and sacrouterine ligament. The amount of vaginal tissue excision was planned according to patients complaints and vaginal stereognosis of the penis during intercourse. Distal vaginal excision (Fig. 1a) was made as a trapezoid shape with the wide base starting just above the hymenal remnants. If the vaginal introitus is tight enough during bimanual examination, then a slim rectangular vaginal tissue was removed instead of a trapezoid shape. Proximal vaginal plate excision was also done in a trapezoid shape with the wide base on the posterior fornix (Fig. 1b). The width of trapezoids was tailored to end up in a two-finger-width vagina after operation. The operation started by dissecting vagina away from the rectum from hymen all the way up to posterior fornix. After removing the vagina, sacrouterine ligament plication was done with Ethibond suture 2/0 and Denonvilliers’ fascia was also pulled up by the same suture by continuous sutures if there were uterine prolapse and constipation symptoms.

Fibromuscular tissue in the outer region of the vagina on either side of the excised vaginal margins was sutured to bring the ends into the midline by continuous uninterrupted no 1/0 polyglactin sutures. Vaginal mucosa and fascia were sutured by 2/0 polyglactin sutures. Both no 1/0 and no 2/0 sutures were kept to continue down to the level of hymen. Perineoplasty and perineal body repair were done if there was any defect. Anterior cervical ring repair and attachment of pubocervicovaginal fascia to the same suture passing from both cardinal ligaments were done whenever there was anterior vaginal wall laxity. A triangle shape vaginal mucosa was de-epithelized and buried beneath this anterior cervical ring repair suture. The mucosa was then closed with 2/0 polyglactin sutures.

Each patient agreed to sign an informed consent to participate the study, and local ethic committee approved the study (Decision Number: 6-01.2023).

The Female Sexual Function Index Questionnaire Turkish version (Validated by Ergün Öksüz) [24] was utilized prior to operations and in the postoperative 6 th month.

Patient satisfaction was evaluated with a Likert scale.

Preoperative demographic characteristics, preoperative POP-Q evaluations, intra- and postoperative complications were recorded.

Statistical analyses were done using SPSS20 (IBM Corp., Armonk, NY). Categorical variables were compared using chi-square test, and continuous variables were compared via paired samples *t* test. A *p* value of <0.05 was considered to be statistically significant.

## Results

All of the 57 patients completed the study. The mean age of the patients was 36.5±5.4 (27–47). The mean gravity was 3.8±2 (0–11), and parity was 3.1±1.8 (0–10). Vaginal delivery rate was 92.9% (53/57). Twenty-two patients (38%) were current tobacco users. After bimanual and speculum examination, 25 (43.9%) of the patients were diagnosed to have anterior vaginal wall laxity, 49 (86%) posterior vaginal wall laxity, and 23 (40.3%) uterine prolapsed with sacrouterine ligament laxity.

All patients were operated with posterior vaginal excision. Forty-nine patients operated on both distal and proximal part of the posterior vaginal wall and eight operated on only proximal part. Anterior cervical ring repair and pubocervicovaginal fascia stretching were added in 25 cases while sacrouterine ligament plication and Denonvilliers’ fascia stretching were added in 23 cases. Perineoplasty was done in 13 cases with episiotomy revision in nine cases.

Early postoperative complications were acute urine retention (*n*=7, 12.3%), vaginal infections (*n*=5, 8.8%), posterior vaginal hematoma (*n*=6, 10.5%), dyspareunia at first intercourse (*n*=22, 38.5%) dyspareunia at 6^th^ month (*n*=5, 8.8%), transient de nova constipation (*n*=11, 19.3%).

Vaginal hematomas were managed conservatively in three cases, and early postoperative revision within six hours of surgery was done in three cases. Vaginal infections were diagnosed by malodorous discharge after surgery managed by metronidazole 1 gr per day for five days. Laxatives were used for constipation. One week of prolonged urinary bladder catheterization was sufficient to overcome the inability to void after surgery. Vaginal water-based lubricants (Neujoys vaginal lubricant, Kocaeli, Turkey) were prescribed to all cases throughout the first six months after surgery.

Satisfaction of the patients from the surgery was classified on a Likert scale so as to: very satisfied 27 (47.4%), satisfied 12 (21.1%), neither satisfied nor dissatisfied 8 (14%), dissatisfied 5 (8.8%), very dissatisfied 1(1.7%). Dissatisfied cases had dyspareunia at the 6^th^ month of surgery and received daily vaginal dilatation therapy. A very dissatisfied case was complaining of laxity and reoperated for further tightening.

The degree of prolapse and presence of pelvic floor complaints prior to surgery and at the 6 th month postoperatively are shown in Table 1.

**Table 1:** Pelvic organ prolapse classification and pelvic floor complaints prior to and after surgery

POP-Q, (%)	Prior to surgery *n*=57	Six months after surgery *n*=57	*p*
0	0	39 (68.4%)	< 0.001**
1	25 (43.9%)	14 (24.6%)	
2	32 (56.1%)	4 (7%)	
Stress urinary incontinence	21(36.8%)	6 (10.5%)	0.001**
Urgency	14 (24.5%)	3 (5.2%)	0.01**
Frequency	7 (12.2%)	0	0.006**
Nocturia	2 (3.5%)	0	0.1
Constipation	16 (28%)	5 (8.7%)	0.007**

**Statistically significant, chi-square test, *p*<0.05

FSFI scores before surgery and at the 6^th^ month postoperatively are shown in Table 2. All the domains and the total score of FSFI were observed to be improved. Only the improvement in the pain domain scores was not statistically significant.

**Table 2:** Follow-up of FSFI after surgery for pelvic organ prolapse

Variable (mean ± SD)	Prior to vaginal tightening *N*=57	Sixth month after vaginal tightening *n*=57	*P*
Desire	3.2 ± 1.1	3.6 ± 1.3	<0.001*
Arousal	3.5 ± 1.4	3.8 ± 1.7	<0.001*
Lubrication	3.4 ± 1.3	2.8 ± 1.4	<0.001*
Orgasm	3.7 ± 1.7	3.3 ± 1.9	<0.001*
Satisfaction	4.0 ± 1.3	3.8 ± 1.3	0.001*
Pain	4.7 ± 1.7	4.5 ± 2	0.3
Total score	23.2 ± 7.3	26.9 ± 5.4	<0.001*
FSFI < 26.5, (%)	35(61.4)	23(40.3)	0.002

*Statistically significant, Paired samples t test, *p*<0.05

## Discussion

Despite the fact that the vagina is a sexual organ, most of the literature pertaining to outcomes of pelvic organ prolapse and incontinence surgery focuses on anatomic rather than functional outcomes. Furthermore, those who mention sexual function usually do not use the same validated tools to assess female sexual function which makes it more difficult to compare the results of different techniques. In general, most of the reports, including a Cochrane review, agree that the repair of prolapse is associated with improvements in sexual function and dyspareunia [25, 26]. Classically, posterior compartment repair is reported to be associated with de novo dyspareunia secondary to levator plication and vaginal narrowing [27]. In the study of Jafarzade and Ulu, it is stated that anterior vaginal repair for symptomatic cystocele may decrease orgasm frequency and intensity with no change in desire or arousal domains while resulting an increase in pain [10]. On the other hand, abdominal approaches such as sacrocolpopexy, sacrohysteropexy, or pectopexy (both laparoscopic and robotic) were reported to be associated with more successful outcomes in sexual function [28, 29].

The posterior vaginal approach for tightening had some advantages based on previous studies. In one vaginal innervation study, distal vagina was found to have more nerve fibers compared to proximal parts and anterior vaginal wall was more densely innervated compared to the posterior wall [15]. Also, in our previous studies 51% of the women reported that on the anterior vaginal wall there is a more sensitive region identified during sexual intercourse or finger stimulation [30]. Furthermore, PRP injection in the lower anterior vaginal wall increased FSFI scores in women [31]. The posterior vaginal wall approaches avoid the densely innervated distal anterior vagina. Also, anterior cervical ring repair with pubocervical-vaginal fascia tightening avoids distal anterior vagina. Tightening the vagina also helps increase the contact of penis with anterior vaginal wall. All these can explain the higher postoperative FSFI scores.

In our study, we excluded the women with stage 3 and 4 prolapse. In a study done by Jelovsek et al., it is noted that women with advanced POP had decreased body image and quality of life [32]. Female sexuality is believed to have multiple dynamics. Among them, psychological drive, self-confidence, and self-contention play a major role. Since correcting advanced stages of POP will not always have to address problems in sexuality directly, it will help to build the women’s self-esteem and provide a better mindset for sex. For this reason, we preferred to include the women with early stages of POP (vaginal laxity), to overcome possible bias.

Our study has limitations because of a relatively small number of subjects and its design which compares pre- and postoperative FSFI scores. It is apparent that larger series that compare different techniques on a specific group of patients (i.e., anterior or posterior vaginal wall prolapse or apical prolapse) are needed to draw more robust conclusions.

In this study, we demonstrated that the sexual function of women with vaginal laxity can be improved when the proposed vulvovaginal erotogenic complex is not disrupted. This can be achieved via a posterior approach while maintaining successful anatomic correction of both posterior and anterior compartments at the same time.

Nevertheless, it can be said that with the increasing number of studies on the way to illuminate the female sexual response, the focus of the surgeries would inevitably shift into obtaining better results in terms of female sexual function.

## Declarations

### Peer-review

Internally peer-reviewed.
